# Building Physician Capacity for HPV Vaccine Uptake in India: Mixed-Methods Implementation Outcomes of a Train-the-Trainer Model Using RE-AIM

**DOI:** 10.3390/vaccines14080654

**Published:** 2026-07-25

**Authors:** Ashleigh Flowers, Shaylen Foley, Swati Saxena, Sutapa Biswas, Priya Ganeshkumar, Purna Kurkure, Upendra Kinjawadekar, Sara Comstock, Nina DaSilva Batista, Meenu Anand

**Affiliations:** 1 American Cancer Society, 270 Peachtree St NW, Suite 1300, Atlanta, GA 30303, USA; shaylen.foley@cancer.org (S.F.); meenu.anand@cancer.org (M.A.); 2 Independent Researcher, New Delhi 110021, India; swati1107@gmail.com; 3 Cancer Foundation of India, 47/2D Selimpur Road, Kolkata 700031, India; sutapa@cficancer.org; 4 Federation of Obstetric and Gynaecological Societies of India, Trade World, C-5,6,7,9,12,13, 1st Floor D-Wing Entrance, Kamala City, Senapati Bapat Marg, Lower Parel (W), Mumbai 400013, India; drpriyagk@gmail.com; 5 Indian Academy of Pediatrics, Kamdhenu Business Bay, 5th Floor, Plot No. 51, Sector 1, Juinagar East, Nerul, Navi Mumbai 400706, India; purna.kurkure@gmail.com (P.K.); upen228@gmail.com (U.K.)

**Keywords:** HPV vaccination, train-the-trainer, physician training, cancer prevention, program evaluation, medical societies, RE-AIM

## Abstract

**Background**: India accounts for one-fifth of global cervical cancer deaths, yet Human Papillomavirus (HPV) vaccination coverage remains low despite the World Health Organization (WHO) recommendations to initiate vaccination at age 9. With the national HPV vaccination campaign in 2026 and its approval for integration into India’s Universal Immunization Programme (UIP), strengthening physician knowledge, beliefs, and confidence in recommending the vaccine is critical. **Methods**: In 2023, the American Cancer Society and Cancer Foundation of India catalyzed two national medical societies, the Federation of Obstetric and Gynaecological Societies of India and the Indian Academy of Pediatrics, to educate physicians on HPV vaccination using a train-the-trainer (ToT) model. A mixed-methods evaluation, using the RE-AIM Framework, included pre- and post-training surveys assessing changes in knowledge, beliefs, confidence, and intent, as well as monthly engagement surveys and project reports analyzed using descriptive and inferential statistics. A fidelity assessment evaluated consistency of training delivery. Twenty in-depth interviews explored physician reactions and implementation of learnings, analyzed using a rapid approach. **Results**: A total of 18,206 physicians were trained. The post-training respondent group demonstrated higher HPV vaccination knowledge scores and more favorable responses in confidence, beliefs, and intent than the pre-training respondent group. The fidelity assessment demonstrated consistent delivery overall, with some variability in role play facilitation. Interviews highlighted increased physician confidence, peer knowledge sharing, and community engagement following the training. Medical societies reported strong program adoption and plans for continued efforts. **Conclusions**: A ToT physician training cascaded through medical societies is a feasible strategy to prepare physicians to recommend the HPV vaccine and address parental concerns at scale.

## 1. Introduction

Globally, there were approximately 661,000 new cervical cancer cases and 348,000 resulting deaths in 2022 [1]. India alone accounted for a staggering 20% (127,000) of new cases and nearly 23% (80,000) of deaths [1,2]. Although cervical cancer rates have decreased slightly over the last few decades in India, rates remain high compared to other countries [3]. Without expanded prevention and detection efforts, the number of individuals who die from cervical cancer in India is projected to nearly double by 2050 [4]. Human papillomavirus (HPV) vaccination can prevent more than 90% of cervical and other HPV-related cancers, and the World Health Organization (WHO) recommends vaccinating girls starting at age 9 [5,6,7]. The vaccine has been available in India since 2008; however, HPV vaccination coverage is low, estimated to be about 6% among the general population [8].

The WHO announced a call for action in 2018 to eliminate cervical cancer by 2030 through three key strategies: fully vaccinating 90% of girls with the HPV vaccine by age 15, screening 70% of women for cervical cancer by the age of 35 and again by 45, and treating 90% of women with pre-cancer and managing 90% of women with invasive cancer [9]. Despite being an extremely cost-effective strategy for cervical cancer prevention, the HPV vaccine faces key challenges that prevent adolescents in India from receiving it [10,11].

Cost and availability of the vaccine are major barriers to uptake [12]. Since 2008, both a quadrivalent and bivalent vaccine have been available in India. A nonavalent vaccine was made available in 2018 [13,14]. A locally produced vaccine, Cervavac, was approved and launched in 2022 [15]. The cost of these vaccines is approximately 3000 rupees (36 USD), ranging from 2000 rupees per dose for Cervavac to over 10,000 rupees for Gardasil 9. These vaccines are only available via prescription by a private practitioner, another barrier to vaccination [16,17].

Studies report that, historically, the cost of the vaccine and its non-availability in the government’s Universal Immunisation Programme (UIP) inhibit physicians from recommending the vaccine to parents [12,18,19]. One study across Southeast Asia found that when the HPV vaccine was offered for free, even if knowledge was poor, parent uptake and acceptance increased [20]. In 2018, following a pilot, Sikkim was the first state in India to offer the HPV vaccine free of cost to all girls aged 9 to 13 statewide. Sikkim implemented two HPV vaccination campaigns through school-based vaccination programs in 2018 and 2019, achieving HPV vaccination coverage of over 95% for the first dose and over 90% for the second dose among girls targeted [14,21]. In 2024, the government of India announced a plan to introduce the HPV vaccine to the UIP for girls aged 9 to 14 to receive it in schools or health facilities at no cost [17,22]. In February 2026, the government rolled out a national HPV vaccination campaign targeting 14-year-olds, delivering a single dose of the vaccine free of cost through government health facilities. Following this campaign, HPV vaccination is approved for integration into the UIP [23,24,25].

Research shows that Indian parents hesitate to accept the vaccine due to concerns of side effects or safety, not perceiving cervical cancer as serious, stigma around the vaccine, and reservations about sexual activity at a young age [12,26,27,28]. Strong provider recommendations have been shown to be a key factor in parental decision making around the vaccine in numerous settings [26,29,30].

Physicians report several challenges to making strong recommendations including a lack of routine wellness visits among the target age group, perceived parental hesitancy, limited knowledge, and low confidence recommending the vaccine even when knowledge and attitudes were high [12,31,32,33]. Studies have also found that physicians lack awareness of HPV vaccination and its importance for cancer prevention [18,19]. Evidence from predominantly high-income countries suggests implementing a multi-component intervention to increase HPV vaccination, including provider and patient education and training, outreach, reminders, and funding [19,34,35]. There has been limited evidence for these interventions in India and other lower- and middle-income countries (LMICs).

With the rollout of a free vaccine and its planned integration into India’s UIP, it is important to address gaps in physician knowledge, attitudes, and beliefs around the HPV vaccine, while increasing physician confidence recommending it.

In 2023, the American Cancer Society (ACS) partnered with the Cancer Foundation of India (CFI) to train and equip physicians with the science of cervical cancer and HPV vaccination, as well as effective communication strategies to use when recommending the vaccine to parents and answering their questions. Two national medical societies, the Federation of Obstetric and Gynaecological Societies of India (FOGSI) and the Indian Academy of Pediatrics (IAP), led and orchestrated implementation of the program. The program used a train-the-trainer (ToT) model and aimed to educate over 20,000 physicians throughout India. This paper uses the RE-AIM framework to present the mixed-methods findings of the cascaded program [36]. Our primary goal was to translate findings of HPV vaccination behavioral research into resources and guides, and then utilize these in a real-world setting to increase physician knowledge, confidence, and recommendation intent related to HPV vaccination in India, while also assessing the successes, reach, and challenges of this implementation.

## 2. Materials and Methods

FOGSI and IAP, in collaboration with ACS and CFI, trained pediatricians and obstetrician–gynecologists (OBGYNs) using a ToT model to support physicians with making effective HPV vaccination recommendations to parents. The following sections detail the training model and evaluation methods used to measure impact of the program.

### 2.1. Formative Research and Translation of Findings to Resources

Prior to the development of the training program, the Center for Social and Behavior Change at Ashoka University led behavioral research to understand the sources of hesitancy among physicians and develop and test behavior change communication solutions to reduce physician hesitancy in recommending the HPV vaccine. Researchers conducted 97 interviews to develop behavioral interventions and then evaluated five interventions using a randomized experiment in a sample of over 600 physicians. Interventions using salience and physician champions significantly increased intention to recommend the HPV vaccine. ACS incorporated the findings into An Action Guide for Medical Societies in India, available at https://preventglobalhpvcancers.org/wp-content/uploads/2023/02/ACS-Action-Guide-India-06.1.pdf (accessed on 21 July 2026) and used the guide as a foundational tool to develop the training [12,37,38].

### 2.2. Collaborative Model

Our collaborative model used a local civil society organization, in this case CFI, to drive implementation work. CFI’s cancer elimination vision and mission in India helped ground this effort and was critical to the successful implementation with medical societies. Medical societies, FOGSI and IAP, then championed and facilitated trainings for member physicians. ACS provided cancer prevention expertise, ToT design support, and implementation funding.

### 2.3. Training Implementation

**Figure 1:**
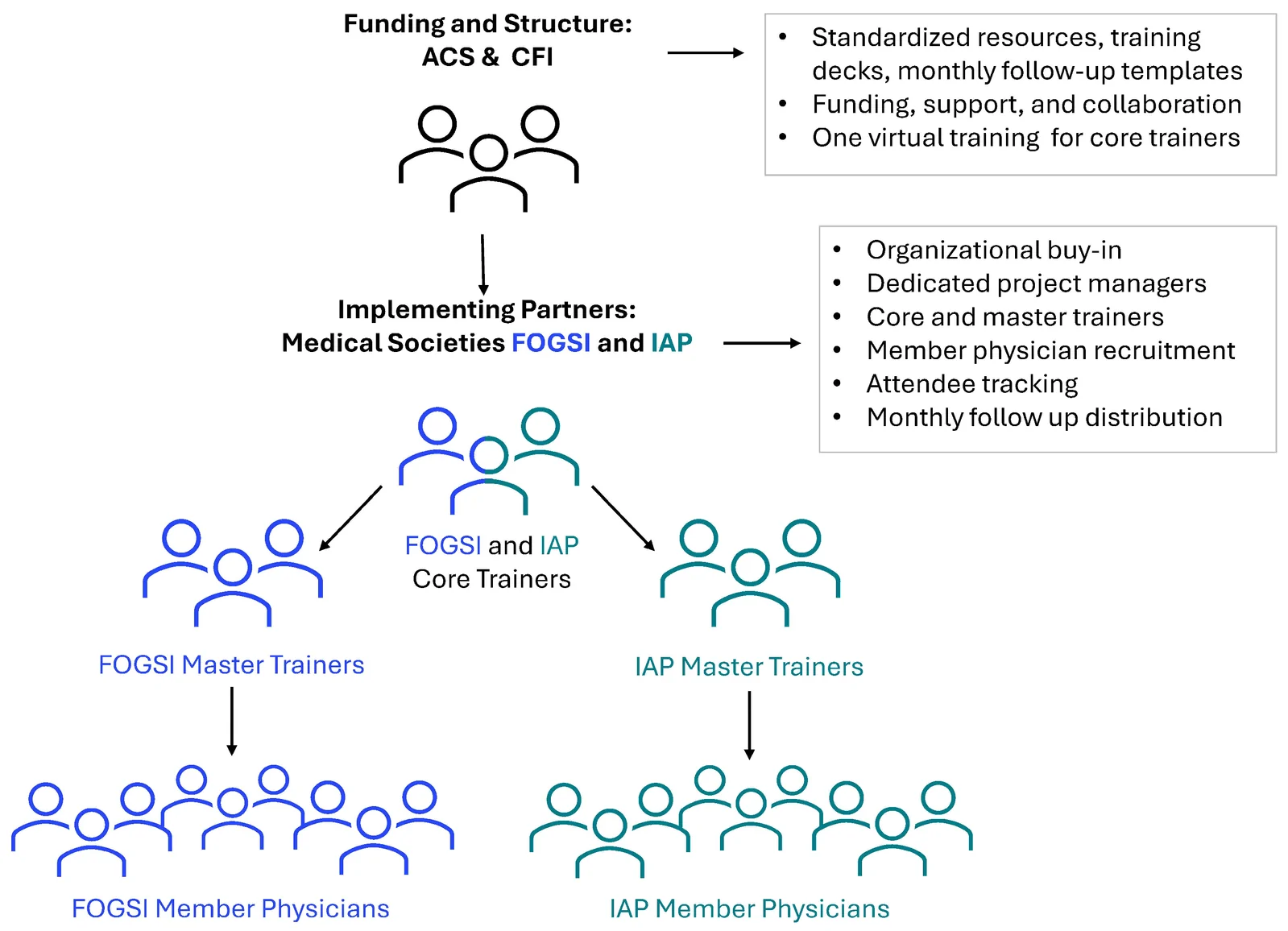
Collaborative model and cascaded train-the-trainer (ToT) structure.

The training aimed to (a) refresh physicians on the cervical cancer burden and HPV vaccination facts, and (b) increase physician confidence in communicating about HPV vaccination by sharing evidence-based messaging and communication strategies. The ToT model cascaded training across three levels—core trainers, master trainers, and member physicians (Figure 1). In September 2023, ACS and CFI trained core trainers during one training session with both FOGSI and IAP physicians. Core trainers then virtually trained master trainers at their respective medical societies. Master trainers proceeded to train member physicians at their medical society using virtual or in-person sessions. Medical society leaders and master trainers recruited member physicians via WhatsApp. The program team implemented the training over 12 months from September 2023 through August 2024.

### 2.4. Curriculum Content

Each training session lasted two hours and was broken into two modules: (1) Cervical Cancer Prevention and HPV Vaccination Facts and (2) Strategies to Effectively Communicate about HPV Vaccination with Parents. Module 1 used standardized PowerPoint decks with pre-recorded presentations by experts in English. Module 2 incorporated role playing to allow physicians to build confidence responding to common questions and concerns around the vaccine. All trainers received a facilitation guide and speaker notes for the slides [39].

### 2.5. RE-AIM Framework

We conducted a mixed-methods evaluation. Results of the evaluation will be presented across the five domains of the RE-AIM framework. RE-AIM domains, data sources, and indicators were mapped out in an evaluation planning matrix (Table 1).

### 2.6. Quantitative Data Collection

Trainers administered pre- and post-surveys at the beginning and end of the training using SurveyMonkey [40]. There were separate surveys for core and master trainers and member physicians. The pre-training survey was anonymous and collected data on demographics and experience with HPV vaccination. The post-training survey collected feedback on the training using two statements on a 5-point scale and one open-ended question. Surveys also measured changes in three knowledge questions and shifts in confidence, beliefs, and intent using six 5-point Likert statements ranging from strongly agree to strongly disagree. Three additional statements on the trainer post-survey measured confidence in training other physicians. Participants who completed the post-survey were able to enter optional contact information to receive completion certificates as a non-monetary response incentive. Because survey responses were anonymous and could not be matched across time points, we were unable to determine how many participants completed both surveys. Therefore, the reported pre- and post-training sample sizes represent respondent groups rather than paired participants. Certificate counts were tracked separately and could not be linked to survey responses.

To encourage and monitor ongoing engagement post-training, both medical societies distributed monthly surveys and activity prompts from November 2023 through June 2024 to participants who attended a training and provided contact information (Table A2). Participants were eligible to complete the monthly survey each month it was distributed. The survey included the same five questions and was disseminated via Google Forms [41]. FOGSI and IAP also submitted end-of-project reports summarizing program reach, adoption, activities implemented, successes and challenges, and overall feedback for CFI and ACS in a Google Document [42].

### 2.7. Quantitative Data Analysis

We used Stata v.18.0 for quantitative data cleaning and analyses and Excel for creating visuals [43]. We exported four pre- and post-survey datasets from SurveyMonkey and used Stata to merge them into one dataset. A subset of master trainers were sent the member physician pre- and post-surveys. We merged these responses into the full dataset but excluded these observations from the analysis of the core and master trainer statements measuring confidence in training other physicians. Our analysis used a pooled dataset due to the inability to match anonymous responses.

We employed descriptive statistics to summarize physician demographics, HPV vaccination experience, and training feedback. To analyze change in HPV vaccination knowledge, we coded the three knowledge questions as correct or incorrect. We additionally created a composite binary variable indicating whether respondents answered all three questions correctly or had at least one incorrect response. We used a Chi-square test using a two-by-two contingency table to assess group-level differences from pre- to post-training in (1) the proportion of correct responses for each knowledge question and (2) the proportion of respondents who answered all three questions correctly. In addition, we calculated Cohen’s h to assess the strength of the training’s impact on knowledge change. To analyze change in participant confidence, beliefs, and intent, we first calculated the proportion of respondents who selected each of the ordinal response categories on the 5-point scale pre- and post-training. We then used a Cochran–Armitage Test for Trend to assess whether there was a significant linear trend in responses across the ordinal categories between pre- and post-training respondent groups. We exported monthly survey data from Google Forms and imported it into Stata to be merged, cleaned, and analyzed using descriptive statistics.

### 2.8. Qualitative Data Collection

One team member based in India conducted twenty in-depth interviews in Spring 2024 to (a) gather data on physicians’ reactions to the content and delivery of the training, and (b) learn how physicians have been implementing what they learned from the training into their clinical practice and community engagement. Interviews included four IAP master trainers, four FOGSI master trainers, six IAP member physicians, and six FOGSI member physicians. We used automatic random selection in Excel to generate a list of participants who we contacted via WhatsApp and phone. If no contact was made, we moved to the next person on the list. The interviewer had no prior relationship with participants, no role in delivering the training, and used a semi-structured interview guide to ensure consistency across interviews while allowing participants to elaborate on their experiences. The interviews were virtual, lasted 30 min to an hour, and were recorded after obtaining consent. All interviews were in English except for one in Hindi. Some interviewees spoke a few sentences in local languages, which we translated verbatim during transcription.

### 2.9. Qualitative Data Analysis

Ongoing reviews and discussions of preliminary takeaways occurred as interviews were conducted. Rev Artificial Intelligence transcribed interviews [44]. The team member who conducted interviews reviewed transcripts for accuracy, spelling of locations, and local jargon and then used a rapid qualitative analysis approach by manually reviewing all transcripts to identify emerging themes related to participant experiences, implementation of training learnings, and recommendations for improvement. The interviewer and two additional team members then discussed all emerging themes, which were compiled with summaries and illustrative quotes in Excel. The group then reviewed the summaries and themes, and findings were refined through discussion and consensus in an iterative process.

### 2.10. Fidelity Assessment

We conducted a fidelity assessment by reviewing video recordings with a structured matrix. We assessed: delivery (how the intervention was delivered and the quality of delivery including the skills, expertise, and knowledge of the trainers and their adherence to good teaching practices); specificity (how well the intervention was designed in terms of gap identification and target trainee selection); engagement (the quality and involvement of trainees); adherence (the extent to which there was consistency in planning, delivery of curriculum, and assessment); and exposure/duration (the extent to which there was variability in training session length and attendance). One team member reviewed nine training sessions provided by program managers from FOGSI and IAP which were sampled to represent different master trainers across multiple geographic zones. Each domain was scored out of five with accompanying qualitative notes. Two additional team members reviewed the scoring sheets and discussed findings throughout the assessment process.

### 2.11. Institutional Review Board

The Morehouse School of Medicine designated this as research not involving human subjects (protocol 2091131-1, obtained 08/2023). The Institutional Ethics Committee of Chittaranjan National Cancer Institute, Kolkata, did a full review and approved the study (protocol CNCI-IEC-SB-2023-82, obtained 10/2023).

## 3. Results

Our findings are presented in order of the RE-AIM domains. Each section presents the corresponding findings and may include a mixture of quantitative and qualitative findings from various sources.

### 3.1. Reach

During the program period, the ToT trained 18,206 physicians, comprising approximately 20% of the total membership across FOGSI and IAP. The team trained 28 core trainers, who trained 510 master trainers, who then trained 17,668 member physicians. Of the member physicians, 14,976 attended at least 70% of the training and received a certificate. Core trainers conducted 12 virtual trainings and one in-person training for master trainers. Master trainers delivered 230 virtual and 160 in-person sessions for member physicians. We received 10,748 pre-surveys and 9173 post-surveys. The majority of participants were female (83.9%) and trained by FOGSI (76.4%) (Table 2). Approximately half of the participants worked in a private hospital (50.0%) and had over 10 years of experience as a physician (57.6%). Participants represented geographically diverse regions of India, with most participants located in the North (32.4%), West (25.2%), or South (21.3%) zones. Trainers delivered the majority of training sessions virtually (64.8%). Pre-training, most participants reported that they had talked about the HPV vaccine with parents (82.4%). Among those who had conversations, participants identified the recommended age for vaccination (57.8%) and side effects (47.8%) as the most common parental concerns. About half of participants reported routinely administering vaccines (48.3%), and most of those participants reported having ever administered the HPV vaccine (83.3%).

### 3.2. Effectiveness

The training was associated with differences in HPV vaccination knowledge between pre- and post-training respondent groups (Table 3). The proportion of correct responses for question one on the cancers caused by HPV was 84.9% correct among pre-training respondents and 96.6% correct among post-training respondents, a difference of 11.7 percentage points (χ^2^ = 773.23, *p* < 0.001, Cohen’s h = 0.43). Correct responses for question two on the youngest age for delivery was 87.7% correct among pre-training respondents and 98.5% among post-training respondents, a difference of 10.8 percentage points (χ^2^ = 856.54, *p* < 0.001, Cohen’s h = 0.47). The proportion of correct responses for the third question on effective communication strategies was 88.7% correct among pre-training respondents and 95.6% correct among post-training respondents (χ^2^ = 311.06, *p* < 0.001, Cohen’s h = 0.26). The percentage of participants answering all three correctly was 70.4% at pre-training and 91.9% at post-training, reflecting a 21.5 percentage point difference (χ^2^ = 1400, *p* < 0.001, Cohen’s h = 0.57). A Cohen’s h of 0.57 indicates a moderate to large effect size, suggesting meaningful differences in knowledge outcomes between pre- and post-training respondents.

**Figure 2:**
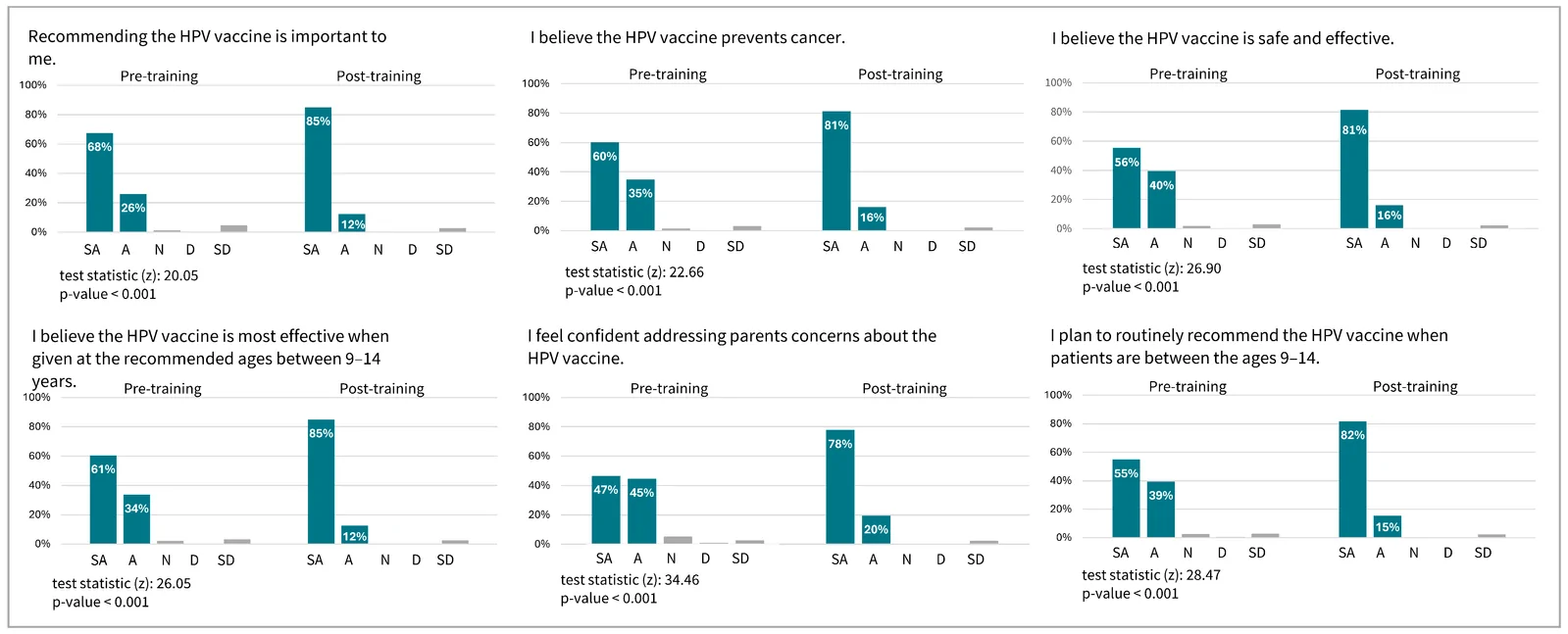
Changes in HPV vaccination beliefs, confidence, and intent among member physicians (pre-training, *n* = 10,748; post-training, *n* = 9173). Likert scale response options: strongly agree (SA), agree (A), neither agree nor disagree (N), disagree (D), and strongly disagree (SD). Test statistics (z) and *p*-values were derived using the Cochran–Armitage test for trend.

The training was also associated with differences between pre- and post-training respondent groups in HPV vaccination confidence, beliefs, and intent, with the post-survey respondent group reporting more favorable responses across all six Likert statements (Figure 2). The largest difference was observed in participants’ confidence addressing parental concerns about the HPV vaccine, which increased 31 percentage points from 47% of pre-training respondents to 78% of post-training respondents *strongly agreeing* (z = 34.46, *p* < 0.001). Trained physicians’ intent to routinely recommend the HPV vaccine at ages 9 to 14 was also more favorable, with 55% *strongly agreeing* pre-training compared to 82% of post-training respondents (z = 28.47, *p*-value < 0.001). Participants’ belief that the HPV vaccine is safe and effective shifted 25 percentage points from 56% of pre-training respondents to 81% of post-training respondents *strongly agreeing* (z = 26.90, *p* < 0.001). Post-training, core and master trainers reported confidence in training peer physicians on the content (96% *strongly agreeing* or *agreeing*) and having time to deliver the training (92% *strongly agreeing* or *agreeing*) (Table A3). The majority of participants rated the training excellent (63.6%) or very good (28.0%) and the content extremely useful (62.1%) or very useful (34.7%) (Table A4).

Two effectiveness themes emerged from interviews that aligned closely with quantitative results: improved confidence and improved communication with parents (Table 4). Physicians described increased confidence recommending the HPV vaccine and a greater ability to address parents’ concerns regarding safety, efficacy, and side effects. Interviewees attributed stronger communication skills to gains in HPV vaccination knowledge and reported more frequently initiating HPV vaccine conversations with mothers of adolescents, older girls, and other patients.

### 3.3. Adoption

#### 3.3.1. Medical Society Collaboration, Leadership Buy-In, and Member Engagement

Medical societies reported that the collaborative model between medical societies, CFI, and ACS provided them with expertise and broadened reach. Leadership within medical societies was also pivotal to engagement and strategic direction. Both medical societies updated HPV vaccination guidelines during implementation. IAP’s changes emphasized ages 9 to 14 years old, advocated for inclusion into the UIP, and highlighted safety and efficacy. Additionally, both FOGSI and IAP reported implementing ongoing activities to encourage member physicians to champion HPV vaccination post-training by recognizing physician efforts and disseminating resources.

#### 3.3.2. Physician Interest, Initiative, and Resource Uptake

Interviews highlighted physicians’ motivation to participate, their engagement in peer outreach, and their use of provided resources. The majority of interviewees reported a prior interest in preventive oncology, research, vaccines, and women’s health. Three of the twenty physicians wished to update their HPV vaccination knowledge, while four wanted to raise community awareness on HPV vaccination and cervical cancer. Nearly half of the physicians interviewed, particularly those in larger practices, reported proactively reaching out to peers, including pediatricians and other OBGYNs, and coordinating in new ways post-training to promote vaccine advocacy within their professional networks.

Training resources, such as brochures and pamphlets, were well received and helped physicians share information quickly and effectively. Both medical societies formed WhatsApp groups to share HPV vaccination resources with trained physicians. Physicians reported translating resources into local languages such as Tamil and Punjabi, displaying them in clinics and hospitals, and forwarding them to parent WhatsApp groups.

### 3.4. Implementation

#### 3.4.1. Training Fidelity During Implementation

The fidelity assessment revealed minimal variability in training content delivery between core trainers and master trainers, as the same PowerPoint slides and pre-recorded didactic components were used across training sessions. Variability in delivery of this portion of the training was limited to instances in which facilitators paused to translate or re-explain content or when internet connectivity issues affected delivery of the pre-recorded components. Greater variability was observed in the role play section of the training, including how trainers provided feedback on participant role plays, the length of time spent on the role play activity, trainee participation levels (e.g., some trainings had substantial engagement and conversation, whereas others had participants with cameras off or that did not respond when called on), and language used. We observed that generally OBGYNs were more engaged during role plays than pediatricians.

#### 3.4.2. Training Acceptability and Recommendations

The training format was well received. Though we saw variation in role play engagement, interviews highlighted widespread appreciation of the role play section, as it encouraged participation and discussion. The main criticism was the length. Virtual and in-person trainings were two hours, which physicians said was a great demand on their time. The length of the preset sections left little time for role play and discussion.

Physicians described the content and materials as very useful, particularly the inclusion of India-specific data. A few physicians suggested that content be included in local languages to make dissemination to the general public easier. Other training content recommendations included:

- A comparison between the vaccines Gardasil and Cervavac;
- Additional details on side effects and their management;
- Help in solving logistical issues like supply bottlenecks;
- More patient management specifics like administration for boys, effectiveness by age, what to do for a missed dose, the spacing between doses, and completing the schedule with different vaccines.

The majority of trainings were virtual. Trainers praised the virtual modality for reaching geographically dispersed practitioners. Interviewees reported in-person sessions had increased engagement, fewer technical issues, and opportunities for connection.

#### 3.4.3. Implementation Adaptations

To address recruitment and participation challenges, changes to implementation occurred at both FOGSI and IAP halfway through the 12-month project, including training a new cohort of master trainers and shortening content from two hours to 90 min. The shorter format was well received and helped improve participation while maintaining the core training content.

### 3.5. Maintenance

#### 3.5.1. Medical Society Sustainability

Both FOGSI and IAP reported plans to sustain efforts beyond the project period. FOGSI designed a second wave of training and stabilization of its Mahila Kavach Kendra, a walk-in vaccination center. IAP reported intentions to conduct awareness campaigns, provide post-training support, recognize champions, leverage local leadership, and advocate for UIP inclusion.

#### 3.5.2. Physician Promotion Activities and Behavior Change

In interviews, physicians described leading awareness generation camps and group discussions on HPV vaccination at schools and colleges, resident associations, religious meetings, melas (fairs), and other cultural congregations post-training.

Monthly surveys highlighted physicians’ adjusted behaviors post-training (Table A5). Respondents were most likely to use communication strategies from the training with parents (85.9%) and share facts within their communities (87.6%). A greater share of pediatricians than OBGYNs reported tangible actions, such as displaying posters (44.1%), sharing pamphlets (52.0%), or posting on social media (50.8%). Most respondents (91.6%) reported conversations about the HPV vaccine with parents of 9 to 14-year-olds post-training. Pediatricians were more likely to report conversations in the last 30 days. Cost was the most frequently selected question from parents (43.6%). The majority of physicians (69.8%) felt prepared to answer questions from parents following the training.

#### 3.5.3. Contextual Factors That EMERGED from Interviews Around HPV Vaccination in India

For long-term maintenance of the program, it is crucial to understand the context in which physicians are operating. Apart from training feedback and use of learnings in their practices, physicians in interviews provided insights into the contextual challenges they face regularly (Table A6). These included the high cost of the vaccine, supply issues in smaller cities, parental fears about side effects, the challenge pediatricians have in reaching girls after age ten, parental lack of awareness in sub-populations, and competing clinical priorities that may take precedence over HPV vaccination.

#### 3.5.4. Physician Suggestions for UIP Roll Out

Almost all interviewed physicians discussed the importance of adding the HPV vaccine to India’s UIP. Physicians believed that rolling out the indigenous vaccine through government channels would significantly improve public trust and uptake, as long as implementation is well-communicated and adequately resourced. They emphasized the need for mass media, celebrity endorsements, and local-language outreach through radio, dramas, or village meetings. Physicians also emphasized the need to train the entire health system at the district and village level, including community-based cadres such as Accredited Social Health Activists, or ASHAs, and Auxiliary Nurse Midwives.

## 4. Discussion

India carries a substantial share of the world’s cervical cancer burden. HPV vaccination effectively prevents cervical cancer and other HPV-related cancers. Recent national policy actions, including the launch of a 3-month free HPV vaccination campaign in 2026 and planned integration into India’s UIP, represent a significant step toward HPV cancer prevention. Our ToT successfully reached over 18,000 OBGYN and pediatricians across India, educating physicians on HPV vaccination science and communication. Two national Indian medical societies adopted this implementation, in collaboration with a cancer civil society organization, to reach a sizable portion of their membership in 12 months. The post-training respondent group demonstrated higher HPV vaccination knowledge scores and more favorable confidence, beliefs, and intent than the pre-training respondent group, reflecting the RE-AIM domain of effectiveness. Physicians reported both applying and maintaining what they learned with patients and peers. Interview feedback on the implementation efforts was positive and included suggestions to improve the length, content, and participation format. Overall, this collaboratively implemented ToT helped prepare Indian physicians to prioritize and confidently recommend the HPV vaccine and laid a foundation that could support national UIP rollout.

ToTs are a common way to cascade educational or implementation efforts for providers and have been used successfully for HPV vaccination in the US [45]. In LMICs, ToTs have been used to educate health care providers during a national rollout of the HPV vaccine in Ethiopia [46]. A review on HPV programs in 46 countries found cascaded training to be less expensive than transporting national experts [47]. Peer trainers may also contribute to increased reach compared to external experts [48].

Providers and health care workers are trusted vaccine messengers [49,50,51,52]. Training physicians, medical students, and other clinical staff on HPV vaccination communication is a common intervention [53,54]. Similar to our ToT, most immunization trainings report outcomes like increased knowledge, shifts in confidence and beliefs, and changes in intention to recommend the vaccine [55]. Building provider confidence and intent to recommend a vaccine is a key social process that can drive patient motivation to receive the vaccine [56]. Additionally, our monthly survey data and interviews suggest that trained physicians used a variety of tactics intended to support HPV vaccination awareness and uptake. Pediatrician respondents were more likely to put up HPV vaccination posters and share pamphlets, while OBGYNs reported peer sharing, vaccination of clinic staff, and community awareness generation activities, suggesting that when given various resources and information, engaged providers cultivate opportunities appropriate to their specialty and clinical setting.

### 4.1. Implementation Lessons Learned and Recommendations

We found collaboration crucial to implementing a ToT of this size. Medical societies, FOGSI and IAP, led implementation. CFI engaged the medical societies, provided high-level program coordination, and solved day-to-day problems. ACS provided funding, strategy, materials, and content expertise. Medical society presidents were key champions, highlighting the importance of leadership and organizational commitment. This partnership led both medical societies to update their HPV vaccination recommendations and develop organizational resources, a sign of long-term commitment to this work.

Master trainers implemented the training with moderate fidelity with observed variations in length, supplemental use of local languages, and the facilitation of role plays. Ongoing assessment allowed us to make small adjustments mid-project to training content and engagement efforts. Insights from implementation of this project may inform policymakers as the Indian government scales HPV vaccination efforts. This project also helped inform subsequent or similar implementation efforts. FOGSI used participant feedback to guide the creation of training material for a second phase [57], which went from January 2025 to March 2026, trained 31,501 physicians, and continued to demonstrate the potential of this ToT model. Findings from this evaluation also informed the development of an asynchronous online HPV vaccination training program, in partnership with the International Pediatric Association, to equip medical societies and healthcare workers with strategies to improve uptake. To date, 13 country medical societies have signed on, with potential reach across more than 140 countries through their membership.

### 4.2. Contextual Factors in India Surrounding HPV Vaccination

We anticipated parental concerns around early sexual debut and fertility. Instead, physicians most commonly reported concerns around cost, side effects, and lack of inclusion in the UIP. We also found cost concerns among providers in our formative qualitative research [12]. LMICs generally have a positive perception of vaccination, with access and cost being the largest initial hurdles to uptake [58]. India’s recent HPV vaccination campaign launch should allow physicians to prioritize HPV vaccination and confidently recommend it more easily. However, successful implementation will likely depend on physician ability to educate parents, address questions about vaccine safety and effectiveness, and counter misinformation. Experiences in other LMICs highlight the importance of government endorsement and promotion to uptake [59,60]. The 2025 rollout in Pakistan serves as a recent example [61]. Interestingly, India’s childhood vaccination rates lag other LMICs due to distrust in the healthcare system and vaccine misinformation, a challenge that may shape coverage even with government backing [62]. This distrust showed up in interviews when providers emphasized the need for mass media campaigns, training of other healthcare cadres, and mitigation of bad press when rolling out the HPV vaccine in the immunization schedule. As vaccine access expands through the UIP, these factors may become increasingly important determinants of vaccine uptake. Physician training remains relevant not only for improving HPV vaccination knowledge, but for preparing physicians to address questions, respond to misinformation, and build public trust in HPV vaccination.

### 4.3. Strengths and Limitations

We implemented this ToT model at a large scale using practical and replicable methods. There are limited examples in peer-reviewed literature of ToTs for HPV vaccination, particularly in LMICs, making this a valuable contribution. Training content was informed by behavioral research and developed with a broad range of stakeholders. Applying the RE-AIM framework strengthened our assessment, as did the use of mixed-methods data collection. We used a structured yet agile approach to implementation, allowing for adaptation. This evaluation primarily assessed implementation outcomes and proximal, self-reported behavioral outcomes. We were unable to assess vaccination uptake and, similar to other physician training evaluations, used intent to recommend vaccination and self-reported numbers of conversations as proxy measures of potential program impact. These measures are subject to recall and social desirability bias, so they cannot be interpreted as direct measures of behavior change. Since our efforts targeted FOGSI and IAP member physicians, trainees practicing in the public sector may have been unable to deliver non-UIP vaccinations immediately post-training and findings may not be generalizable to all health care worker groups involved in HPV vaccine delivery. We were also unable to assess whether the training reached physicians serving underserved populations. Participation in the post-training and monthly surveys was voluntary and may overrepresent physicians who were more engaged with HPV vaccination activities. As this was a real-world implementation at scale, our training design did not contain a control group or use an experimental design. Additionally, because our survey responses could not be matched across time points, quantitative findings reflect group-level comparisons between pre- and post-training respondent groups and should be interpreted alongside the broader mixed-methods findings.

### 4.4. Recommendations

Future implementation efforts should aim to measure vaccination uptake following training. This ToT content and model could be adapted to train general practitioners, India’s community health workers, known as Accredited Social Health Activists, and other front-line health workers to support and bolster recommendations from physicians. Additional versions of resources in local languages could strengthen utilization and reach. Subsequent training should also consider incorporating cervical cancer screening content with HPV vaccination to support efforts toward cervical cancer elimination.

## 5. Conclusions

The HPV vaccine prevents cervical cancer and is a planned addition to the UIP in India. A large-scale ToT physician training cascaded with the leadership of two medical societies is a viable approach for supporting physician engagement in HPV vaccination efforts. The post-training respondent group demonstrated higher HPV vaccination knowledge scores and more favorable confidence, beliefs, and intent than the pre-training respondent group, while interviews suggested that participants applied training learnings with patients and peers. These findings demonstrate favorable implementation outcomes. The ToT was strategically implemented to precede the current rollout of the HPV vaccine nationally in India.

## Figures and Tables

**Table 1:** Evaluation Matrix using RE-AIM Framework.

Domain	Data Sources	Indicator
Reach ^1^	Program Records	Number of physicians trained
	Pre-Post Survey	Number of physicians who received a completion certificate
		Demographics of participants
		Participant level of HPV vaccination experience
Effectiveness ^1^	Pre-Post Survey	Change in HPV vaccination knowledge ^2^
	Qualitative Interviews	Change in HPV vaccination confidence, attitudes, beliefs ^3^
		Proportion reporting satisfaction with the training
		Qualitative themes around impact of the training on knowledge, confidence, attitudes, and beliefs
		Qualitative participant feedback
Adoption	**Medical Society:**	**Medical Society:**
	End of Project Reports	Qualitative feedback around medical society adoption
		
	**Physician:**	**Physician:**
	Qualitative Interviews	Motivation to participate
Implementation ^1^	Fidelity Assessment	Barriers and facilitators to training implementation
	Qualitative Interviews	Qualitative feedback about training delivery
	Program Records	Adaptations made during implementation
Maintenance	**Medical Society:**	**Medical Society:**
	End of Project Reports	Qualitative feedback around continued/future work
		
	**Physician:**	**Physician:**
	Qualitative Interviews	Qualitative feedback around engagement and implementation
	Monthly Surveys	Proportion reporting champion activities and parent recommendations

^1^ These three domains reflect only individual level (physician) indicators. ^2^ For response options, see Appendix A Table A1. ^3^ Assessed using six Likert-scale statements: (1) Recommending the HPV vaccine is important to me; (2) I believe the HPV vaccine prevents cancer; (3) I believe the HPV vaccine is safe and effective; (4) I believe the HPV vaccine is most effective when given at the recommended ages between 9–14 years; (5) I feel confident addressing parents’ concerns about the HPV vaccine; and (6) I plan to routinely recommend the HPV vaccine when patients are between the ages 9–14.

**Table 2:** Participant Demographics, Training Details, and Experience ^1^.

		Total		Pediatrician		OBGYN		Other	
Variable, *n* (%)		*n* = 10,748		*n* = 1508		*n* = 8749		*n* = 491	
**Medical Society**									
	IAP	1584	(14.7)	1473	(97.7)	70	(0.8)	41	(8.4)
	FOGSI	8209	(76.4)	3	(0.2)	7978	(91.2)	228	(46.4)
	Other	955	(8.9)	32	(2.1)	701	(8.0)	222	(45.2)
**Training Group**									
	Core/Master Trainers	459	(4.3)	267	(17.7)	187	(2.1)	5	(1.0)
	Member Physicians	10,289	(95.7)	1241	(82.3)	8562	(97.9)	486	(99.0)
**Gender**									
	Male	1720	(16.0)	875	(58.0)	706	(8.1)	139	(28.3)
	Female	9022	(83.9)	633	(42.0)	8038	(91.9)	351	(71.5)
	Other	2	(0.02)	0	(0)	1	(0.01)	1	(0.2)
	Prefer not to say	4	(0.04)	0	(0)	4	(0.1)	0	(0)
**Zone**									
	North	3487	(32.4)	328	(21.8)	2916	(33.3)	243	(49.5)
	South	2289	(21.3)	384	(25.5)	1824	(20.9)	81	(16.5)
	East	1510	(14.1)	282	(18.7)	1164	(13.3)	64	(13.0)
	West	2704	(25.2)	280	(18.6)	2344	(26.8)	80	(16.3)
	Central	758	(7.1)	234	(15.5)	501	(5.7)	23	(4.7)
**Workplace Type**									
	Private hospital	5375	(50.0)	693	(46.0)	4437	(50.7)	245	(49.9)
	Private clinic	1812	(16.9)	372	(24.7)	1374	(15.7)	66	(13.4)
	Public hospital	3527	(32.8)	487	(32.3)	2916	(33.3)	124	(25.3)
	Public clinic	87	(0.8)	17	(1.1)	63	(0.7)	7	(1.4)
	Other	825	(7.7)	114	(7.6)	628	(7.2)	83	(16.9)
**Years of Experience**									
	0	1449	(13.5)	187	(12.4)	1049	(12.0)	213	(43.4)
	1 to 4	1853	(17.2)	232	(15.4)	1552	(17.7)	69	(14.1)
	5 to 9	1255	(11.7)	188	(12.5)	1047	(12.0)	20	(4.1)
	10 or more	6191	(57.6)	901	(59.8)	5101	(58.3)	189	(38.5)
**Training Location**									
	In person	3781	(35.2)	520	(34.5)	3014	(34.4)	247	(50.3)
	Virtual	6967	(64.8)	988	(65.5)	5735	(65.6)	244	(49.7)
**Talked about HPV vaccine with parents**									
	Yes	8851	(82.4)	1207	(80.0)	7312	(83.6)	332	(67.6)
	No	1897	(17.7)	301	(20.0)	1437	(16.4)	159	(32.4)
**Main questions/concerns about HPV vaccine**									
	Recommended age	6215	(57.8)	877	(58.2)	5113	(58.4)	225	(45.8)
	Side effects	5138	(47.8)	627	(41.6)	4323	(49.4)	188	(38.3)
	Fertility concerns	2285	(21.3)	266	(17.6)	1897	(21.7)	122	(24.8)
	Other	702	(6.5)	179	(11.9)	496	(5.7)	27	(5.5)
**Routinely administer vaccines**									
	Yes	5186	(48.3)	1128	(74.8)	3858	(44.1)	200	(40.7)
	No	5562	(51.8)	380	(25.2)	4891	(55.9)	291	(59.3)
**Administered the HPV vaccine** ^2^									
	Yes	4323	(83.3)	884	(78.4)	3342	(86.6)	97	(48.3)
	No	865	(16.7)	244	(21.6)	517	(13.4)	104	(51.7)

^1^ Data were obtained from the pre-survey. ^2^ This question was only asked of respondents who answered “Yes” to “Routinely administer vaccines.”

**Table 3:** Participant HPV Vaccination Knowledge Change ^1^.

	Pre-Training	Post-Training	% Point			
	% Correct	% Correct	Increase	Chi^2^	*p*-Value	Cohen’s *h*
Q1: HPV can cause which of the following cancers?	84.9%	96.6%	11.7	773.23	<0.001	0.43
Q2: What is the youngest age it is recommended to start delivering the HPV vaccine?	87.7%	98.5%	10.8	856.54	<0.001	0.47
Q3: Effective communication strategies for talking to parents about the HPV vaccine are:	88.7%	95.6%	6.9	311.06	<0.001	0.26
**All three questions correct**	70.4%	91.9%	21.5	1400	<0.001	0.57

^1^ Sample sizes were *n* = 10,748 for the pre-survey and *n* = 9173 for the post-survey.

**Table 4:** Train-the-Trainer Program Qualitative Results by RE-AIM Domain from Physician Interviews (*n* = 20) and Medical Society Project Reports (*n* = 2).

Domain	Theme	Level	Description	Quotes
Effectiveness				
	Improved confidence	Physician	Physicians reported increased confidence in recommending the HPV vaccine after the training.	“Now we can confidently tell this vaccination to the parents. We can counsel the parents. Previous to the training, we ourselves were in a dilemma like what would be the side effects, and what [are] all the implications, and what is the effectiveness?” (Gynecologist) “Most of my patients had already read about [the HPV vaccine]. My role was to reassure and clarify.” (Gynecologist)
	More effective communication with parents	Physician	Physicians reported increased confidence in recommending the HPV vaccine after the training.	“We start by saying, ‘Even I vaccinated my daughter.’ That reassures them.” (Gynecologist) “They’re [parents] interested, they are becoming more and more aware. So there are questions which parents of adolescent children are asking us about the HPV vaccination. So it is a good thing that we are trained in explaining to them the basics and the need for the vaccine. And I’m experiencing a good response from the parents and they are accepting the facts also.” (Pediatrician) “No, it’s not myths, I think they’re just not aware that it [HPV vaccination] is required [for cancer prevention]. Once I told them, within seven days, they got their daughters vaccinated. I had to tell them that it’s important, it’s safe.” (Gynecologist) “We were vaccinating before also [with HPV], but now it has become more rigorous… we are explaining to the patients, the mothers, about their children.” (Gynecologist)
Adoption				
	Collaboration and medical society leadership buy-in	Medical society	FOGSI and IAP described adoption by the leaders at their medical society and collaboration with the other stakeholders as key factors to success.	“The medical society is proud of its collaboration with partners who shared a common goal of increasing HPV vaccination rates. These partnerships played a key role in the success of the training program and helped extend the reach of important information to a broader audience.” (Medical society representative) “The involvement of partner organizations, ACS and CFI, brought in crucial expertise that helped refine the project’s approach.” (Medical society representative) “Committed leadership played a pivotal role in guiding the online HPV vaccination training program to success. Leaders within the medical association demonstrated unwavering dedication to the cause, provided clear direction, and inspired participants to actively engage in the training. Their vision and support created a foundation for excellence and instilled confidence in the project’s objectives.” (Medical society representative)
	Decision to participate	Physician	Most physicians admitted having a prior interest in HPV infection, cervical cancer and the HPV vaccine, and several desired more knowledge on the topic.	“Actually, I have had a niche interest in the preventive oncology side. So I felt the field of preventive oncology is really the need of the hour in this time. So HPV vaccination really it’s one of a boon.” (Gynecologist) “Main reason [to attend the training] is the need to enlighten myself on what is new... The parents are quite vigilant in asking about HPV and all. So it is always better if we get some latest insight.” (Pediatrician)
	Peer outreach	Physician	Physicians described proactively reaching out to peers and coordinating in new ways to promote HPV vaccine advocacy within their professional networks.	“I have coordinated with my pediatrician colleagues also that they’re counseling children up to 14 years who are not coming to me.” (Gynecologist) “Luckily my wife is also obgyn specialist. I coordinated with her and also with other obgyn specialists. I also advise all the other gynecologists of the town to give this vaccine.” (Pediatrician)
	Resource utilization	Physician	Physicians used HPV vaccination resources within their clinics or hospitals and disseminated them to patients.	“Yeah, it’s [HPV vaccination posters] in my clinic. I have a couple of these I have made and got printed on a A4 size poster.” (Pediatrician) “I got it [HPV vaccination resource] translated from my residents and made charts as I told you... I also sent it across to all the people who I had trained and they in the clinics have put it up for the knowledge.” (Gynecologist) “Yeah, we did use those sheets...we translated into Tamil because that would be more beneficial to them [local patients]. And we also use the other pamphlets. I have some of the pamphlets displayed in my clinic also.” (Pediatrician)
Implementation				
	Length of the training sessions	Physician	Trainers and participants felt that the training length was too long and a great demand on their time.	“I believe content was good, but yeah, it was very lengthy because obviously the attention span of people won’t last for such a long time.” (Pediatrician) “Videos took a long time though. We had interaction time, but that time was less. If we had more time for interaction, that would’ve been better. “ (Gynecologist)
	Role play section	Physician	Role play was appreciated as it encouraged participation and discussions.	“In both my training sessions we did do role play... previously, we had a talk with the respective doctors, and we discussed what has to be done in the role play. It is very informative.” (Gynecologist) “See, role plays session is always useful because there are certain sets of question which are asked by the parents... So when we do a role play we come across various types of question and various types of answer given the various doctors.” (Pediatrician)
	Training content	Physician	Training content was well received, particularly the inclusion of India-specific data, which physicians found valuable despite familiarity with some HPV content due to specialty. Some respondents suggested content be made more localised and made available in local languages.	“The advocacy material was very good. Especially, it is better for people who are just starting [to advocate for HPV vaccination]. I am already very active into it, so didn’t help me much. But the team whom I trained, I trained around 60, 70 people after that, so they found it helpful.” (Gynecologist) “The training was really absolutely perfect, and in fact I learned many new things, and I got my inquiries cleared. It was really good.” (Pediatrician) “See, we are very highly educated. It is very easy to educate the people who are educated. You have to have a program [and resources] for the lay population.” (Gynecologist)
	Online versus in-person training sessions	Physician	Many trainers praised the virtual modality for reaching geographically dispersed practitioners. However, in-person trainings were better received due to increased engagement and minimal technical issues.	“Online was pretty good. I had a lot of attendance because of the online format. Doctors from all over could attend.” (Gynecologist) “Yeah, because online I don’t know how many participants are there, how many of them attended, how many of them are just on their mobile or they’re really watching it. But, of course, in offline session because the audience are in front, we know.” (Pediatrician)
Maintenance				
	Plans to continue efforts	Medical society	Both FOGSI and IAP reported plans to continue efforts to increase uptake of HPV vaccination after program completion.	“We will conduct regular campaigns using social media, newsletters, and webinars to emphasize the importance of HPV vaccination and reach a broader audience of healthcare professionals.” (Medical society representative) “Physicians will have access to continued support through dedicated resources, materials, and consultations to help them effectively promote HPV vaccination in their practices.” (Medical society representative)
	Novel community awareness opportunities	Physician	Many physicians held awareness generation activities on HPV vaccination post-training.	“We have approached few schools and we have even organized few cervical cancer screening camps also.” (Gynecologist) “We have a pharmacy group where in a monthly meeting there’ll be at least 300 participants, together with the postgraduates from all the medical colleges, from the department of obgyn.” (Gynecologist) “...we are giving awareness to the students and also to the school teacher, because unless the school teachers ask the students to take [HPV] vaccination, they don’t listen to the parents and also to the doctors. Always the best person is the teacher, they [students] follow the teacher’s advice.” (Pediatrician) “Sometime we do the health camps. Myself will go talk about the [HPV] vaccination.” (Pediatrician)

## Data Availability

The data sets presented in this article are not readily available due to privacy concerns. Requests to access deidentified datasets or evaluation tools should be directed to ashleigh.flowers@cancer.org.

## Abbreviations

The following abbreviations are used in this manuscript:

ACS	American Cancer Society
CFI	Cancer Foundation of India
FOGSI	Federation of Obstetric and Gynaecological Societies of India
HPV	Human Papillomavirus
IAP	Indian Academy of Pediatrics
LMIC	Lower- and Middle-Income Countries
OBGYN	Obstetrician-gynecologists
RE-AIM	Reach, Effectiveness, Adoption, Implementation, Maintenance
ToT	Train-the-Trainer
UIP	Universal Immunization Program
WHO	World Health Organization

## Appendix A

**Table A1:** HPV Vaccination Knowledge Questions and Response Options.

Question	Response Options ^1^
Q1. HPV can cause which of the following cancers?	a. Cervical b. Oropharynx c. Penile, vaginal, vulvar, and anal **d.** **All of the above**
Q2. What is the youngest age it is recommended to start delivering the HPV vaccine?	**a.** **Age 9** b. Age 12 c. Age 14 d. Age 18
Q3. Effective communication strategies for talking to parents about the HPV vaccine are:	a. Be brief and direct b. Talk about the HPV vaccine as cancer prevention c. Start recommendation by noting child’s age d. Answer parent’s questions using brief, fact-based messages **e.** **All of the above**

^1^ Correct response option is in bold.

**Table A2:** Training materials and resources.

Timing	Component	Audience	Resources and Monthly Action Prompts
Prior to training	Training Deck and Trainer Guidance	Core and master trainers	Trainers Guide ^1^ Member Training Slides, accessible at https://preventglobalhpvcancers.org/resources/presentation-for-physicians-how-to-counsel-parents-on-hpv-vaccination/ (accessed on 21 July 2026) ^2^
During training	Pre-post survey	Core and master trainers	Core and Master Trainer Pre-Post Surveys, accessible at https://preventglobalhpvcancers.org/wp-content/uploads/2026/04/India-HPV-Vaccination-Training-Program-Trainer-Surveys.pdf (accessed on 21 July 2026)
	Pre-post survey	Member physicians	Member Physician Pre-Post Surveys, accessible at https://preventglobalhpvcancers.org/wp-content/uploads/2026/04/India-HPV-Vaccination-Training-Program-Member-Surveys.pdf (accessed on 21 July 2026)
After training	Member action prompts ^3^	Core and master trainers Member physicians	Monthly Action Prompts and Resources, accessible at https://preventglobalhpvcancers.org/wp-content/uploads/2026/05/Monthly-Action-Prompts.pdf (accessed on 21 July 2026)

^1^ This document is a living resource and has been updated since its use in this program. ^2^ Not publicly available. ^3^ ACS developed monthly email or WhatsApp prompts that included suggested actions, a survey link, and relevant resources. Content was shared with medical society program managers to disseminate to all member physicians who had completed the training prior to the email date.

**Table A3:** Core and master trainer confidence in delivering training to other physicians (*n* = 605) ^1^.

Variable, *n* (%)	SA ^2^		A		N		D		SD	
Confidence delivering training	455	(75.2)	125	(20.7)	6	(1.0)	0	(0)	19	(3.1)
Time to deliver training	360	(59.5)	197	(32.6)	24	(4.0)	7	(1.2)	17	(2.8)
Reviewed trainer checklist & other resources	363	(60.0)	183	(30.3)	33	(5.5)	8	(1.3)	18	(3.0)

^1^ Data were obtained from the core and master trainer post-survey. ^2^ Likert scale response options: Strongly agree (SA), Agree (A), Neither agree nor disagree (N), Disagree (D), Strongly Disagree (SD).

**Table A4:** Participant ratings of training satisfaction and content usefulness (*n* = 9173) ^1^.

Variable, *n* (%)			
**Overall Rating**			
	Poor	3	(0.03)
	Fair	48	(0.5)
	Good	727	(7.9)
	Very good	2564	(28.0)
	Excellent	5831	(63.6)
**Usefulness of Content**			
	Not at all useful	2	(0.02)
	A little useful	28	(0.3)
	Moderately useful	271	(3.0)
	Very useful	3179	(34.7)
	Extremely useful	5693	(62.1)

^1^ Data were obtained from the post-survey.

**Table A5:** Monthly survey results on post-training participant engagement and HPV vaccination promotion activities.

			Total ^1^		FOGSI		IAP	
Variable, *n* (%)			*n* = 1501		*n* = 1255		*n* = 246	
**In the last month I have championed the HPV vaccine in the following ways:**								
	used communication strategies from the training with parents							
		Yes	1289	(85.9)	1076	(85.7)	213	(86.6)
		No	42	(2.8)	36	(2.9)	6	(2.4)
		Not yet—I plan to next month	152	(10.1)	125	(10.0)	27	(11.0)
		No response	18	(1.2)	18	(1.4)	0	(0)
	the “You would do anything for your daughter...” poster is displayed in my clinic							
		Yes	662	(44.1)	536	(42.7)	126	(51.2)
		No	234	(15.6)	200	(15.9)	34	(13.8)
		Not yet—I plan to next month	555	(37.0)	476	(37.9)	79	(32.1)
		No response	50	(3.3)	43	(3.4)	7	(2.9)
	shared the “Don’t wait to vaccinate” pamphlet about HPV vaccination with parents							
		Yes	781	(52.0)	626	(49.9)	155	(63.0)
		No	226	(15.1)	202	(16.1)	24	(9.8)
		Not yet—I plan to next month	450	(30.0)	389	(31.0)	61	(24.8)
		No response	44	(2.9)	38	(3.0)	6	(2.4)
	posted about HPV vaccination on my social media							
		Yes	762	(50.8)	615	(49.0)	147	(59.8)
		No	270	(18.0)	235	(18.7)	35	(14.2)
		Not yet—I plan to next month	423	(28.2)	366	(29.2)	57	(23.2)
		No response	46	(3.1)	39	(3.1)	7	(2.9)
	shared facts about HPV vaccination within my community							
		Yes	1315	(87.6)	1111	(88.5)	204	(82.9)
		No	51	(3.4)	40	(3.2)	11	(4.5)
		Not yet—I plan to next month	112	(7.5)	86	(6.9)	26	(10.6)
		No response	23	(1.5)	18	(1.4)	5	(2.0)
**In the last month I was able to talk to the following number of parents about the HPV vaccine:**								
		None; I hope to find an opportunity next month	127	(8.5)	114	(9.1)	13	(5.3)
		1–5 parents	657	(43.8)	595	(47.4)	62	(25.2)
		6–10 parents	331	(22.1)	256	(20.4)	75	(30.5)
		11–15 parents	150	(10.0)	109	(8.7)	41	(16.7)
		>15 or more parents	236	(15.7)	181	(14.4)	55	(22.4)
**What are the main types of questions you’re hearing from parents about HPV vaccination?** ^2^								
		Safety	551	(36.7)	451	(35.9)	100	(40.7)
		Side Effects	609	(40.6)	513	(40.9)	96	(39.0)
		Fertility	211	(14.1)	185	(14.7)	26	(10.6)
		Cost	654	(43.6)	524	(41.8)	130	(52.9)
		Doses/Schedule	523	(34.8)	425	(33.9)	98	(39.8)
		Age	446	(29.7)	373	(29.7)	73	(29.7)
		Is it required/mandatory?	586	(39.0)	472	(37.6)	114	(46.3)
**Do you feel prepared to answer questions you are getting from parents?**								
		Yes—I feel prepared	1048	(69.8)	874	(69.6)	174	(70.7)
		No—I do not feel prepared	19	(1.3)	14	(1.1)	5	(2.0)
		No response	434	(28.9)	367	(29.2)	67	(27.2)

^1^ Data represents cumulative responses across all months the survey was disseminated. ^2^ November surveys included this as an open-ended question. Those responses are excluded from reporting.

**Table A6:** Contextual Themes from Physician Interviews (*n* = 20).

**Theme**	**Description**	**Quotes**
Cost	Cost was the most cited barrier to HPV vaccination uptake, particularly in semi-urban and rural areas.	“Cost is a major issue in this region. Even educated patients hesitate.” (Gynecologist) “There is a cost issue... the quadrivalent is Rs 2000 and nonavalent is Rs 9000 or 10,000, even after doctor concession.” (Gynecologist) “Yeah, it’s [cost] an issue with some patients. So for them maybe I give them the Serum Institute vaccine, which is less costly.” (Pediatrician) “Since the cost is very high and I don’t think many people can actually afford it, even after explaining it to them and they agree to it, but since the cost is so much, many people don’t actually go for the [HPV] vaccine because it’s an optional thing and it’s not in any immunization schedule... Most people who went for it are all my staff members, my colleagues, people who are in medical setting... and I think some affluent families.” (Pediatrician)
Supply	Supply, while not a major issue in metropolitan areas, was a constraint in smaller towns where providers needed to coordinate with pharmaceutical representatives to ensure availability.	“When patient asks for it [the HPV vaccine] beforehand, I call the pharmacy.” (Gynecologist) “I don’t stock the [HPV] vaccine in the hospital since it’s not yet on the government list.” (Gynecologist) “I keep few doses [of the HPV vaccine] with me of every brand so that whenever the patient arrives, I give the patient right then and there.” (Pediatrician)
Misconceptions	Misconceptions and fears among parents, especially regarding fertility and sexual behavior, were not as widespread as anticipated but still relevant.	“The only thing is they’re asking about are what are the side effects in general. Related to fertility, there is not any specific question.” (Gynecologist) “Most of the time first thing comes to their mind is why are you talking of cervical vaccine and why you are giving it at such a young age. That is the usual question. So there again we have to tell them that sooner is the better.” (Pediatrician) “[Parents ask] whether this vaccine can impose early menopause or whether it’ll reduce the chances of fertility or something like that in the future. These are the usual questions and what we usually say is that this vaccine has been there [in other countries] since 2006.” (Pediatrician)
Age group and coordination	Pediatricians noted that clinic visits and vaccination follow-up decline after age five, leading to missed opportunities for later vaccines.	“Even till ten years of age we are giving [regular scheduled vaccines], but after that [parents] think my child is healthy, so repeated reminders are needed [for additional vaccines like HPV given at ages 9–14].” (Pediatrician) “So general observation is that after five years most of the parents just keep the immunization report aside and during this period [after five years of age] rarely children [are] required to come to our clinic also.” (Pediatrician) “And I start my counseling right from the age of five years... because the next vaccination is after five years, at the age of 10 years...I don’t think there is any method by which we can call the parents or call the patients at 10 years of age because there’s a huge database.” (Pediatrician)
Resistance	Some resistance remains in lower-income groups and less-educated segments, especially when patients are not actively seeking preventive care.	“Usually educated patients don’t have any misconceptions. It’s the lower strata, and we have to counsel them that this is not like this. There is no toxicity in the vaccine… Otherwise, see I’m into private practice and most of my patients are from an educated background. Yeah. So no, I usually don’t see such problems in my practice.” (Gynecologist)
Prioritization	In some smaller cities, physicians admitted that they were still struggling with issues like safe childbirth and basic immunisations, so HPV vaccination is lower in priority.	“We are fighting for the basics only like those concerned with the childbirth. So we are involved in this so much and by the end of the day we are tired. So explaining, dealing with this subject [HPV vaccination] becomes a little negligible…this subject comes a little bit lower down [in priority].” (Gynecologist) “I agree that there are a lot of issues... but we can’t solve all the issues before starting the [HPV] vaccine... I guess those who are capable, they’re taking the vaccines so what is the problem? So I think it should be the priority for all the doctors.” (Pediatrician)
Platform for inclusion into UIP	Physicians across geographies and sectors welcomed inclusion of the HPV vaccine in the UIP, noting that clear communication, adequate resources, and mass IEC campaigns will be critical to building trust and uptake.	“If the government makes it [HPV vaccination] part of the schedule like polio or Hepatitis B, they [parents] will not miss the immunisation schedule.” (Gynecologist) “Whenever government gives out or rolls out a program, I don’t think there will be much resistance unless there is going to be an outbreak of side effects; in that case only there will be a problem.” (Gynecologist) “[There will need to be] mass media coverage of the counseling that will be required because I’m not talking about a one-to-one patient thing… When we will implement it [a new vaccine] for all things like for oral polio vaccine, we have to get a lot of mass media coverage to bring about that acceptability. Same would need to be done for HPV. Otherwise we may face this [resistance] because you have to tell them that this is necessary, this is safe, with the advantages coming out in the form of pamphlets, brochures, newspapers, everywhere.” (Gynecologist) “I think before we roll out the [HPV] vaccination, there’s a lot of training that is very important, you should [train] the NGOs also, that’s also very useful.” (Pediatrician) “I think it [HPV vaccination] should be the priority for all doctors. At the same time I think that the government should take it in the UIP.” (Pediatrician)
Further training of health staff needed	Physicians noted that there was a need to disseminate the training to the entire health system, especially at the district and village level, and to ASHAs and ANMs for rural rollout.	“We took a special session to train ASHAs and MO in VIA screening. So they did very well. [The HPV] vaccine should come in UIP as early as possible [for rural set-up].” (Gynecologist) “The training should go to the periphery also as much as possible to touch everybody, all doctors, all nurses also must know.” (Gynecologist)
